# Rapid Domoic Acid Depuration in the Scallop *Argopecten purpuratus* and Its Transfer from the Digestive Gland to Other Organs

**DOI:** 10.3390/toxins12110698

**Published:** 2020-11-03

**Authors:** Gonzalo Álvarez, José Rengel, Michael Araya, Francisco Álvarez, Roberto Pino, Eduardo Uribe, Patricio A. Díaz, Araceli E. Rossignoli, Américo López-Rivera, Juan Blanco

**Affiliations:** 1 Facultad de Ciencias del Mar, Departamento de Acuicultura, Universidad Católica del Norte, Coquimbo 1281, Chile; jose.rengel@ucn.cl (J.R.); falvarezsego@gmail.com (F.Á.); roberto.pino@alumnos.ucn.cl (R.P.); euribe@ucn.cl (E.U.); 2 Centro de Investigación y Desarrollo Tecnológico en Algas (CIDTA), Facultad de Ciencias del Mar, Universidad Católica del Norte, Coquimbo 1281, Chile; mmaraya@ucn.cl; 3 Doctorado en Acuicultura, Programa Cooperativo Universidad de Chile, Universidad Católica del Norte, Pontificia Universidad Católica de Valparaíso, Coquimbo 17811421, Chile; 4 Centro i∼mar & CeBiB, Universidad de Los Lagos, Puerto Montt 557, Chile; patricio.diaz@ulagos.cl; 5 Centro de Investigacións Mariñas (Xunta de Galicia), Apto. 13, 36620 Vilanova de Arousa, Pontevedra, Spain; araceli.escudeiro.rossignoli@xunta.gal; 6 Laboratorio de Toxinas Marinas, Instituto de Ciencias Biomédicas, Facultad de Medicina, Universidad de Chile, Santiago 1027, Chile; amlopez@med.uchile.cl

**Keywords:** ASP, amnesic shellfish poisoning, detoxification, Northern Chile, toxicokinetics, modeling

## Abstract

Domoic acid (DA), the main toxin responsible for Amnesic Shellfish Poisoning, frequently affects the marine resources of Chile and other countries across the South Pacific, thus becoming a risk for human health. One of the affected resources is the scallop *Argopecten purpuratus*. Even though this species has a high commercial importance in Northern Chile and Peru, the characteristics of its DA depuration are not known. In this work, the DA depuration was studied by means of two experiments: one in controlled (laboratory) and another in natural conditions. All organs of *A. purpuratus* depurated the toxin very quickly in both experiments. In some organs, an increase or a very small decrease of toxin was detected in the early depuration steps. Several models were used to describe this kinetics. The one that included toxin transfer between organs and independent depuration from each organ was the model that best fit the data. It seems, therefore, that the DA in this species is quickly transferred from the digestive gland to all other organs, which release it into the environment. Physiological differences in the two experiments have been shown to have some effect on the depuration from each organ but the actual reasons are still unknown.

## 1. Introduction

Domoic acid (DA) is a naturally produced algal toxin that accumulates in shellfish and is responsible for causing amnesic shellfish poisoning (ASP) [1]. The first intoxication in humans occurred in Prince Edward Island, Canada, in 1987, where more than 100 people became ill after consuming blue mussels [2]. The main symptoms of this intoxication include nausea, gastroenteritis, and vomiting, followed by neurological signs such as confusion, lethargy, disorientation, paresthesia, short-term memory loss, and in extreme cases, death (reviewed by [3]). Since the report of the first outbreak detected in Canada produced by the diatom *Pseudo-nitzschia multiseries* [2], episodes of this type of toxicity have been recorded in many areas around the world (reviewed in [4,5,6]).

In northern Chile, within the framework of the Molluscan Shellfish Safety Program of the National Fisheries and Aquaculture Service (SERNAPESCA), elevated levels of DA have been detected in shellfish from many of the primary aquaculture sites [7]. In some cases, the DA concentrations exceeded the regulatory limit (20 mg kg^−1^), and the harvesting of scallops from aquaculture sites was banned [8,9]. The longest and most intense ASP episode detected thus far occurred in the scallop culture area of Bahía Inglesa, in the austral spring of 2006. The bloom associated with this event was dominated by the DA-producing diatom *Pseudo-nitzschia australis* which reached a maximum concentration of 1.6 × 10^6^ cells L^−1^ (80% of the total phytoplankton biomass) on November 2, 2006. DA concentrations in the scallops were as high as 103 mg kg^−1^ wet weight, the maximum concentration of DA measured in scallops (*Argopecten purpuratus*) from this area (reviewed in [10]). During *Pseudo-nitzschia* blooms, scallops can accumulate sufficient DA to exceed the regulatory limit [11]. In bivalve tissues, the accumulation process depends on several factors, such as the presence and ingestion of toxic *Pseudo-nitzschia* species, the cell toxin content [4], as well as the balance of the physiological mechanisms regulating DA accumulation and depuration [12,13,14,15]. Depuration is the main process regulating maximal DA concentrations in shellfish and how long after the shellfish have ceased feeding on toxic phytoplankton before they are safe for human consumption [13,16,17,18,19].

Numerous studies have been undertaken to develop improved strategies for mitigating the negative consequences of ASP outbreaks. Among this are studies to determine accumulation and depuration of DA in different shellfish. This data are critical for developing kinetic models that allow government monitoring agencies, producers, or fishers to predict how fast shellfish can became toxic, and afterwards, how they will remain toxic [17,20,21]. Two depuration models have generally been used to describe the depuration process in bivalves. The first model is a one-compartment model characterized by an exponential decrease of DA content throughout the entire depuration period. This model has been shown to describe the depuration kinetics of the mussel *Mytilus edulis* adequately [22], and the scallops *Placopecten magellanicus* [15], and *Pecten maximus* [23]. The second model corresponds to a two-compartment model, characterized by rapid toxin elimination in the initial phase of depuration followed by a period of slower toxin elimination. This type of depuration has been reported in the mussels *Volsella modiolus* [24], *Mytilus galloprovincialis* [12], the razor clam *Siliqua patula* [25,26], the surf clam *Mesodesma donacium* [17], and the oyster *Crassotrea virginica* [13].

Other studies have been focused on developing specific treatments to reduce or eliminate the toxins from edible tissues of shellfish considering their inter-individual and the anatomical distribution of DA [17,23,27,28,29,30,31,32,33]. In the case of *Pecten maximus*, a species that retains domoic acid for a substantial period of time [23,27,34], studies on the anatomical distribution of DA allowed the implementation of an exemption to the general regulation of the European Community for toxins in bivalve mollusks (European Decision 2002/226/EC), which made harvesting and marketing eviscerated scallops affected by domoic acid possible under special conditions. For this species the DA concentration in the entire mollusk must be less than 250 mg kg^−1^ and less than 4.6 mg kg^−1^ in each of the edible tissues (gonad and adductor muscle) [35,36].

The scallop *Argopecten purpuratus* (Lamarck, 1819) is an endemic species of the Pacific coast of South America. It is distributed from Paita (5°S) in northern Peru to Valparaíso (33°S) in Chile [37,38], dwelling in shallow (<30 m depth) sandy bays [39]. Currently, this scallop is one of the most important commercial species on international markets because of its high nutritional properties [40], relatively large size and fast growth rate [41,42], and high price [43]. Currently, Peru is the third most important scallop aquaculture producer worldwide, behind China, while Chilean scallop production stagnated in the 2000s and recently decreased [41,43,44].

Even when several toxic outbreaks of DA have been detected in the primary *A. purpuratus* aquaculture sites in Northern Chile and presented a potential risk for Peruvian scallop aquaculture, no information is available on depuration of this toxin. In this work the kinetics of domoic acid depuration from different organs of *A. purpuratus* were studied. Mathematical models involving depuration and toxin transfer between organs were developed and implemented to understand the processes involved in depuration and to gain predictive capability.

## 2. Results

Two DA depuration experiments were carried out using scallops which had fed on a naturally occurring bloom of toxic *Pseudo-nitzschia australis*. In the first experiment, scallops were removed from the field, held in the laboratory, and fed non-toxic *Isochrysis galbana* (Haptophyta). Changes in body weight and tissue-specific DA concentration were then measured for 12 days. In the second, experiment the scallops were maintained in the field and allowed to feed naturally on a post-bloom phytoplankton population containing only a relatively small population of *Pseudo-nitzschia australis*.

### 2.1. Experiment of Depuration of Domoic Acid in Controlled Conditions

#### 2.1.1. Weight

**Figure 1:**
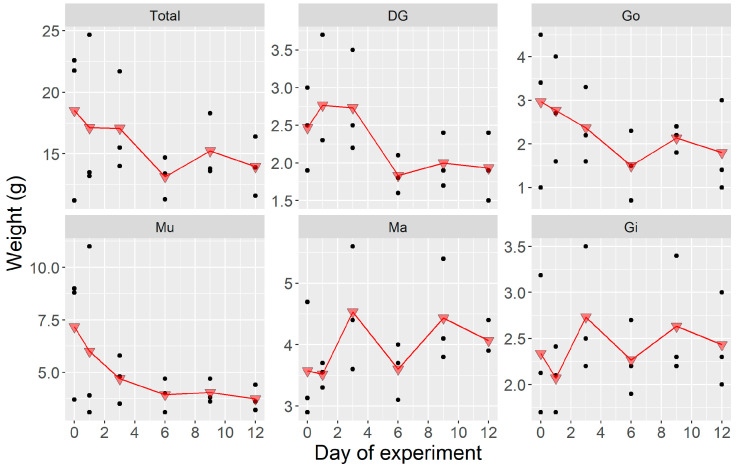
Weight of organs during the laboratory depuration experiment where *Argopecten purpuratus* were fed only the non-toxic *Isochrysis galbana*. Dots are the observed weights (*n* = 3) and triangles are their corresponding means. Total = all soft tissues, DG = digestive gland, Go =gonad + foot, Mu = adductor muscle + kidneys, Ma = mantle, Gi = gills.

During the first laboratory experiment, there was a general, and statistically significant (*p* = 0.04), weight decrease, which took place in some of the body fractions studied. It was observed in DG (digestive gland), Mu (adductor muscle plus kidneys), and Go (gonad plus foot). The biomass of Ma (mantle) and Gi (gills) remained approximately constant throughout the experiment (Figure 1).

#### 2.1.2. Toxin Burden

**Figure 2:**
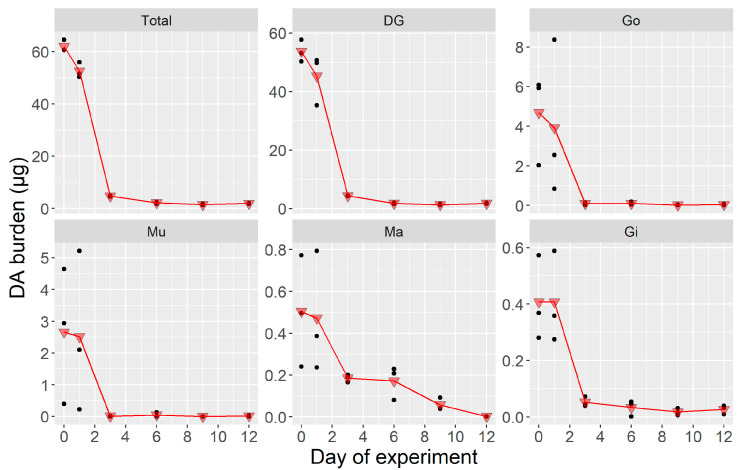
Tissue-specific domoic acid (DA) burden in the laboratory depuration experiment where *Argopecten purpuratus* were fed only the non-toxic *Isochrysis galbana*. Dots are the observed burden (*n* = 3), and triangles are their corresponding means. Total = all soft tissues, DG = digestive gland, Go = gonad + foot, Mu = adductor muscle + kidneys, Ma = mantle, Gi = gills.

Between day 0 and day 1, the whole body of the scallops subjected to the laboratory conditions lost a small percentage of its toxin burden. In some organs, the loss was very small (Mu, Ma) or even nonexistent (Gi). After day 1, the burden decreased abruptly to residual levels in all organs but the mantle, where the decrease was more gradual (Figure 2). Only residual amounts of DA remained by day 3 to 6, depending on the organ. This rapid decrease in DA burden made it impossible to estimate the depuration rate using the data from the whole time series (Figure S1). Instead, the data from the first three days of sampling were used to calculate the depuration rate by fitting a first-order exponential decrease (Figure S2). The rate obtained was 0.91 day^−1^ (which is equivalent to 60% loss day^−1^). The depuration from the different organs ranged from 0.3 day^−1^ for Ma to 2.0 for Mu. Meanwhile, Gi, DG, and Go (in increasing order) had intermediate depuration rates (0.7, 0.9, and 1.5, respectively).

#### 2.1.3. Whole Body and Tissue-Specific DA Concentrations During the Laboratory Experiment

**Figure 3:**
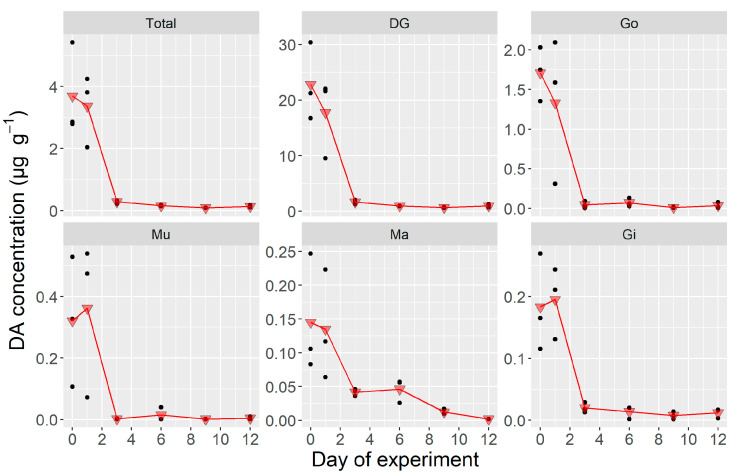
Organ-specific DA concentration in the laboratory depuration experiment where *Argopecten purpuratus* were fed only non-toxic *Isochrysys galbana,* dots are the observed burden (*n* = 3) and triangles are their corresponding means. Total = all soft tissues, DG = digestive gland, Go = gonad + foot, Mu = adductor muscle + kidneys, Ma = mantle, Gi = gills.

The changes in the tissue DA concentrations observed during the laboratory experiment were very similar to those of the burden. The concentration, both in the whole body and the individual organs, decreased quickly to residual levels (Figure 3). The concentration increased or was maintained from day 0 to day 1 in Gi and Ma (which lost almost no weight), but also in Mu. In the Go, Ma, and Mu, the decrease measured in day 6 was smaller than that which could be expected from the concentration recorded in day 3. The estimated rate for the whole body was 0.9 day^−1^. For the different organs, it ranged from 0.4 day^−1^ in the mantle to 1.7 in Mu, with intermediate rates for Gi, DG and Go (in ascending order).

### 2.2. Experiment of Domoic Acid Depuration in the Natural Environment

#### 2.2.1. Weight

**Figure 4:**
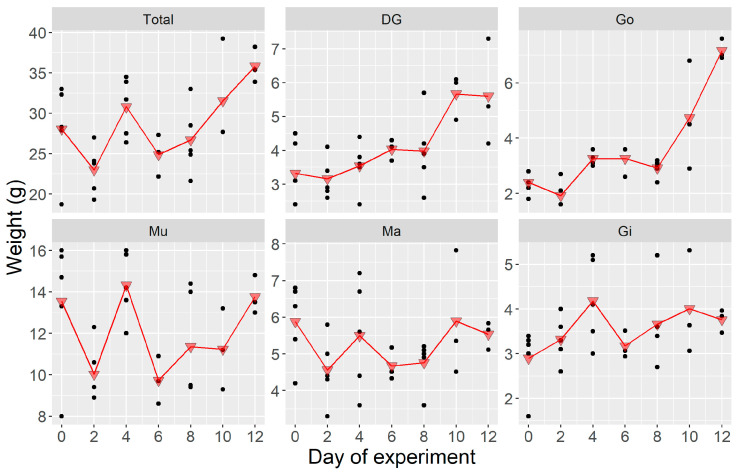
Weight of *Argopecten purpuratus* organs during the depuration experiment where shellfish were incubated in the field under natural conditions. Dots are the observed weights, and triangles are their corresponding means. Total = all soft tissues, DG = digestive gland, Go = gonad + foot, Mu = adductor muscle + kidneys, Ma = mantle, Gi = gills.

During the experiment carried out in natural conditions, there was a general, but non-significant (*p* = 0.07) weight increase (Figure 4). Like in the laboratory experiment, the weight of Ma and Gi remained approximately constant, but those of Go and DG underwent changes, although in this experiment by contrast with the laboratory experiment, their weight increased. The increase was especially pronounced in Go (*p* < 0.0001). Mu maintained its weight throughout this experiment.

#### 2.2.2. Toxin Burden

**Figure 5:**
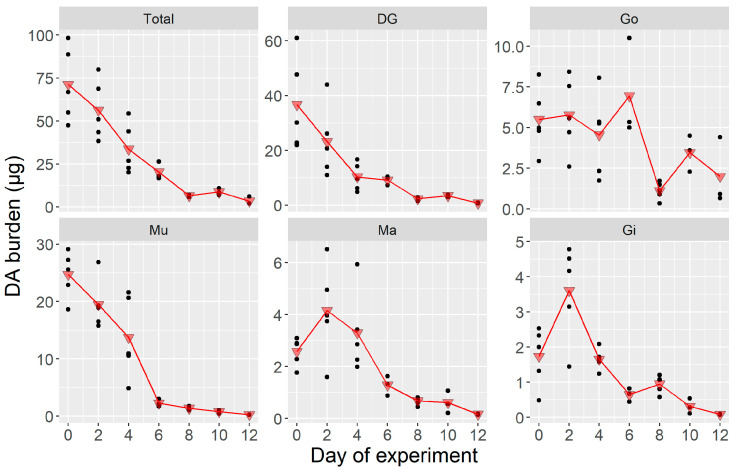
Domoic acid burden of *Argopecten purpuratus* organs during the depuration experiment where shellfish were maintained under natural conditions. Dots are the observed burden and triangles are their corresponding means. Total = all soft tissues, DG = digestive gland, Go = gonad + foot, Mu = adductor muscle + kidneys, Ma = mantle, Gi = gills.

In natural conditions, the total DA burden continuously decreased with time in a nearly negative exponential way (Figure 5) (linear when logarithmically transformed (Figure S3 Supplementary Materials). This decrease was driven by DG and Mu, while Ma and Gi increased their DA content from day 0 to day 2, and the gonad approximately maintained it until day 6. After day 2, the burden decreased in all organs but Go.

Scallops’ depuration in these conditions was also fast, but not as fast as in the laboratory experiment. The whole body depurated at a rate (assuming the same kinetics as for the lab experiment) of 0.27 day^−1^ (equivalent to 24% day^−1^). The recorded differences between organs were also smaller, ranging from 0.12 day^−1^ for Go to 0.40 day^−1^ for the Mu. Gi, Ma, and DG had intermediate depuration rates (Figure S3, Supplementary Materials), with the first two depurating in a very similar way and more slowly than DG. Those estimates were similar to the ones obtained in the laboratory experiment with the main difference being the slower depuration of Go.

#### 2.2.3. Whole Body and Tissue-Specific DA Concentrations During the Field Experiment

**Figure 6:**
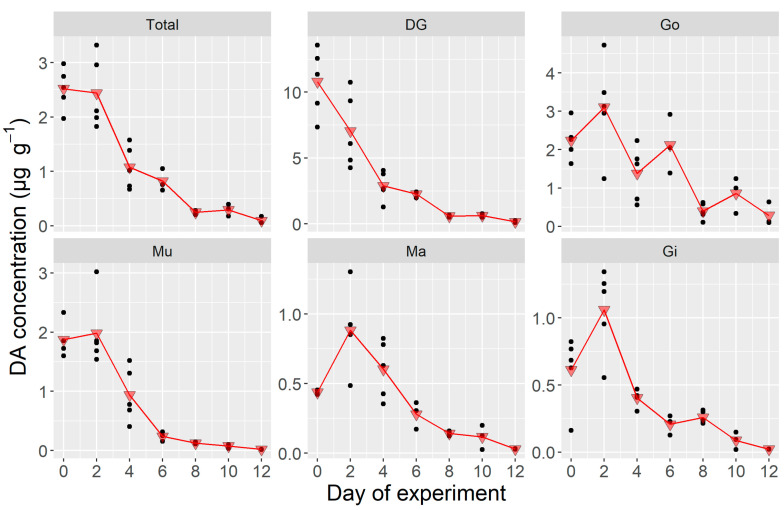
Organ-specific DA concentration in the field depuration experiment where *Argopecten purpuratus* were maintained under natural conditions. Dots are the observed burden, and triangles are their corresponding means. Total = all soft tissues, DG = digestive gland, Go = gonad + foot, Mu = adductor muscle + kidneys, Ma = mantle, Gi = gills.

In the scallops depurated in natural conditions, the concentration of the whole body decreased in a first-order exponential way (linearly when logarithmically transformed (Figure S4, Supplementary Materials), but the observed level for day 2 was slightly higher than expected. The DG concentration (once transformed) decreased linearly without any noticeable deviation in day 2. In Gi, Ma, and Go, notwithstanding, the concentration increased during that day, and in Mu, it was maintained (Figure 6).

The whole body depurated at a rate of 0.29 day^−1^, substantially more slowly than in the experiment carried out in the laboratory. Mu was the body fraction with the highest depuration rate (0.40 day^−1^) and Go, the one with the lowest rate (0.20 day^−1^). The other organs had intermediate rates, depurating DG noticeably faster than the two other organs (Figure S4, Supplementary Materials).

### 2.3. Model Fitting

#### 2.3.1. Depuration of Domoic Acid in the Natural Environment

**Figure 7:**
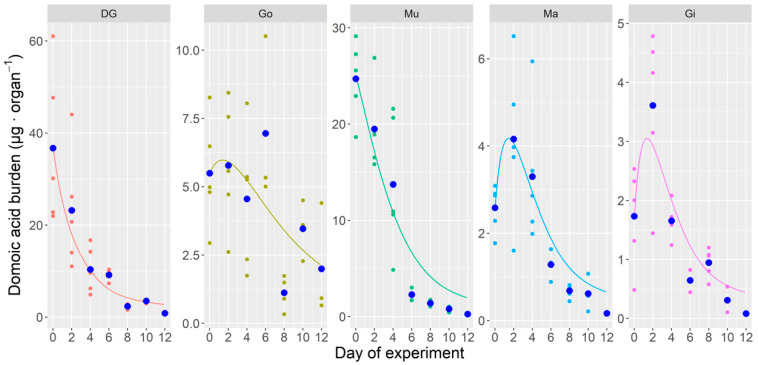
Multi-compartment model fit to the toxin burden data obtained in the DA depuration experiment in natural conditions. Small dots are the obtained data, large dots are their means, and lines are the output of the model. DG = digestive gland, Go = gonad + foot, Mu = adductor muscle + kidneys, Ma = mantle, Gi = gills.

Two types of models were fitted to the experimental data of the toxin burden. The first one was first-order exponential decrease of each organ, without toxin transfer between organs, and the second included toxin transfers from DG to all other organs, which depurated in the same way than in the first type of model. When fitted to the data from the experiment carried out in natural conditions (the complete dataset) the model without transfers of DA from the digestive gland to other organs was unable to correctly describe the depuration, especially because of the increase of toxin burden in the second day of the experiment in some organs. When the transfer between organs was included, the model fits the observed data much better (Figure 7). The simplest model of this type, with only a common transfer rate to all organs and a common depuration rate, described the general shape of the kinetics but did not fit well the levels of DA in several organs, especially in DG (with levels noticeably underestimated).

When different depuration rates were allowed for the digestive gland and all other organs, a better fit was obtained. The improvement, notwithstanding, was not large. The new model only fit slightly better the data of DG and Ma, with no improvement in the other tissues. When the transfer rates and the depuration rates were allowed to vary, the fitting improved, but some aspects of the kinetics were still not correctly described. The DA levels in DG are slightly underestimated, and the final part of the depuration curve of Mu is overestimated. The actual fitting of the Go cannot be correctly evaluated because of its high dispersion. The lowest depuration rate estimated by the model is that for DG, and the two highest were those of Gi and Ma. The transfer rates of the toxin were also the highest for those two organs (Table 1).

#### 2.3.2. Depuration of Domoic Acid in Laboratory

**Figure 8:**
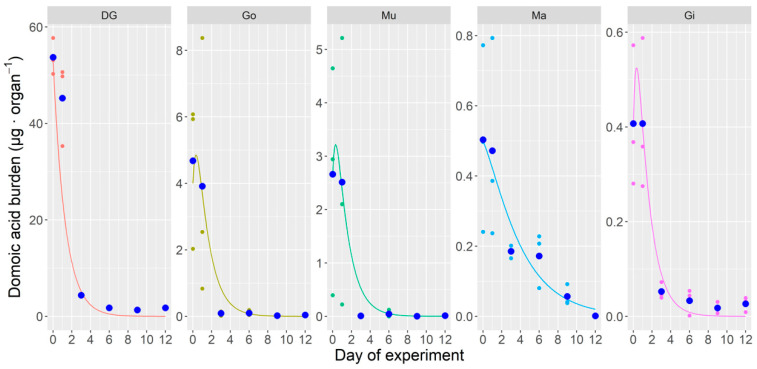
Multi-compartment model fit the toxin burden data obtained in the domoic acid depuration experiment done in the laboratory. Small dots are the obtained data; large dots are their means, and lines are the output of the model. DG = digestive gland, Go = gonad + foot, Mu = adductor muscle + kidneys, Ma = mantle, Gi = gills.

The same model fitted to the data obtained in the laboratory gave different estimates, but the amount of useful observations was very low because of the steep toxin burden descent (Figure 8). The model estimates some transfer of toxin, but the organs to which the toxin transfer was estimated to be higher did not coincide with those in the experiment carried out in natural conditions (Table 1). Some of the observed differences could be explained if the depuration of the different organs does take place totally or partially through the kidney; unfortunately, it was not separated from the adductor muscle in any of the experiments. This may account for the increase of DA in Mu (adductor muscle plus kidney) during the initial steps of the laboratory experiment, in which the depuration rate was very high.

## 3. Discussion

### 3.1. Physiological Conditions in the Two Experiments

During the experiment carried out in the laboratory, where the food availability was approximately 120 mm^3^ day^−1^, the scallops underwent weight loss. Some organs were not affected by that decrease (gill and mantle), but all the others were, especially the adductor muscle and the digestive gland. The resources stored in several organs were therefore used to maintain their metabolism. Scallops usually accumulate nutrient reserves during periods of somatic growth when food is abundant. Among them, glycogen in adductor muscles is the principal source of energy, but protein stored in the same organ and lipids in the digestive gland also play important roles during starvation periods (reviewed by [45]). The opposite situation took place in the scallops from the natural environment. In this case, the abundance of phytoplankton was high (>1,000,000 cell L^−1^) and the estimated food availability was much higher than in the laboratory experiment, with an average of 3200 mm^3^ day^−1^. As a consequence, the food was enough not only for maintenance but also to support some growth and gonadal development.

### 3.2. Domoic Acid Depuration

In both experiments, domoic acid depuration was very fast, independently from how it was measured (decrease of toxin burden or concentration). This species should, therefore, be considered to be a fast depurating one, as is also the case for several mytilids, such as *Mytilus edulis*, *M. galloprovincialis*, *M. californianus*, and other species like the clam *Mya arenaria* [24]. In the laboratory experiment, after the fast-initial decrease of DA, the scallop organs maintained a very low amount of the toxin until the end of the experiment. Residual amounts have been also found in other species whose depuration follows a two-compartment kinetics, like *Volsella modiolus* [24], *Placopecten magellanicus* [20], *Mytilus galloprovincialis* [12], *M. edulis*, *Crassostrea virginica* [13], and *Mesodesma donacium* [17].

There were important differences in the apparent depuration rate between the two experiments. Scallops in laboratory depurated faster than in natural conditions (0.91 vs. 0.27 day^−1^, respectively). Most of the difference is probably due to some degree of reintoxication in natural conditions, because the toxic algae populations had not completely disappeared (Table S1, Supplementary Materials). The two experiments also differed at least in temperature and food availability, and both could have contributed to the observed difference. The difference in the temperature at which the experiments were developed (16 °C in the laboratory and 13–14 °C at natural conditions) could have contributed to the difference in depuration rate, since temperature seems to accelerate the DA depuration in other species, like the king scallop *Pecten maximus* [27] or the detoxification of paralytic shellfish toxins in the visceral mass of the surf clam *Spisula solidissima* [46]. The magnitude of the recorded difference seems, nevertheless, too high to be the only factor, as the estimated Q10 (temperature coefficient) is near 6, while, for most physiological rates in bivalves it is between 2 and 3 [47,48]. Food availability would not seem to be primarily responsible because in sight of its likely negative effect on depuration in the king scallop, *Pecten maximus* [27]. Nevertheless, its effect cannot be ruled out because the physiological differences between the two species could be important.

### 3.3. Domoic Acid Depuration Kinetics

The depuration kinetics in the two experiments were not the same. The toxin levels in the laboratory experiment dropped very quickly to a residual level (less than 0.5% of the regulatory limit) which was maintained until the end of the experiment with small variations. A two-compartment model for each organ can therefore describe it. However, from a practical point of view, the second compartment would be negligibly small, and the kinetics could be described by an initial (3 days) exponential decrease and a subsequent stabilization at nearly residual levels. The same situation was observed by Mafra Jr et al. [49].

In natural conditions, the depuration rate was slower (not extremely fast) and the residual levels which characterized the final part of the depuration period were not reached. The kinetics of the DA in each organ can therefore be described by a one-compartment model. The generally good fitting of a straight line to the logarithmically transformed DA burden and concentration indicates that the depuration follows a first-order exponential decrease. Even when the presence of toxic phytoplankton and the consequent re-intoxication could have been expected to alter the exponential kinetics, no effect can be appreciated. The most likely reason is that the decrease of the toxic phytoplankton during the experiment was also approximately exponential (with a decrease rate of 0.17 day^−1^) (Table S1, Supplementary Materials).

The depuration of some organs did not completely follow the expected curve in either experiment. The toxin in some organs did not decrease at the expected rate or even increased between the first and second days of the experiment. As DA is acquired mostly with the ingested phytoplankton, it seems that a quick redistribution of the toxin among all organs took place. The multi-compartmental model fitted to the data, allowing the transfer from the digestive gland to all other organs and the independent depuration of each of them, describes the main characteristics of the observed depuration very well. This suggests that the hypothesized transfer actually takes place and that the depuration from the organs is very fast. With the obtained data it is not possible to discern whether the depuration from the organs took place directly or by transfer in the hemolymph to excretory organs, such as the kidney. It seems, nevertheless, that the later could be the main depuration route for several reasons. First, it is unlikely that the high depuration rate of Mu is due to the adductor muscle, which has a low surface/volume ratio, and consequently, a low rate of exchange with the surrounding water. Second, a multicompartment model similar to Model II (2), but modified to simulate the kinetics of the adductor muscle and the kidney separately, and by assuming that all depuration takes place only through the digestive gland and the kidney, fit much better with the data of Mu in the experiment carried out in natural conditions. However, with that model the fittings obtained for the digestive gland were worse that the ones obtained with Model II (2), indicating that some aspects of the kinetics have not been correctly implemented and suggesting that new experiments should be carried out to unequivocally ascertain the actual excretion route.

The fast depuration rate estimated for Mu could be explained by the fact that the adductor muscle contains a large amount of hemolymph in venous sinuses and an important artery [50]. This organ is in fact the most adequate one to extract hemolymph from bivalves [51]. DA transfer from visceral to other tissues through the circulatory system has been suggested in the oyster *Crassostrea gigas* [52].

Currently, little information exists regarding the distribution of DA from the digestive gland (or visceral tissues) to different organs in other bivalve species. The information that exists indicates this distribution process is a species-specific characteristic. Mafra et al. [13] suggested that the faster DA elimination in visceral tissues of *M. edulis* and *C. virginica* could be explained by toxin transfer to other tissues. In the king scallop *Pecten maximus* DA redistribution seems to be very limited, and restricted to post-spawning periods [23]. In other species, such as the scallop *Placopecten magellanicus,* [20] and the mussel *M. edulis* [13] the toxin has a preferential accumulation in the digestive gland, and there is no evidence if the DA exchange among tissues is relevant in toxin elimination. On the contrary, the anatomical distribution in other species suggests that the redistribution could be an important process in some cases. In *C. virginica,* during the initial days of the toxin accumulation phase a large proportion of DA can be redistributed from visceral to other tissues [13]. Similarly, during the uptake phase in the surf clam *Mesodesma donacium*, toxin was evenly distributed among the soft tissues [17].

Finally, considering the high depuration rate, the time in which the bivalves are unsafe for consumers once the toxic algae populations have disappeared is very short, and therefore the economic losses that could result by the ASP outbreaks in the scallop aquaculture industry should be moderate.

## 4. Conclusions

*Argopecten purpuratus* is a rapid domoic acid depurator (less than 3 days to depurate 50% of the toxin). The toxin acquired by the digestive gland was quickly transported to other organs, which in some cases increased its DA content during the initial phases of the depuration. The depuration of the DA from all organs seems to approximately follow a first-order exponential decay, but a residual amount of the toxin was slowly depurated. It seems very likely that most of the excretion takes place through the main excretory organs (digestive gland and kidney). Physiological conditions seem to affect the depuration kinetics, but the way in which they act is unclear. Domoic acid should not be expected to have a relevant impact on the culture and exploitation of this species, unless toxic *Pseudo-nitzschia* blooms are very persistent in time.

## 5. Materials and Methods

### 5.1. Depuration of Domoic Acid in Controlled Conditions

Scallops (*Argopecten purpuratus*) naturally contaminated with ASP toxins were donated by Invertec Ostimar Company from a farm located in Bahía Tongoy during a *Pseudo-nitzschia australis* bloom detected at the end of July 2017. A sample of 40 commercial size individuals (shell height: 7.73 ± 4.10 cm) was collected and transported to the laboratory within a few hours after collection. Once in the laboratory, the scallops were distributed into six groups of five individuals. Each group was randomly assigned to one day of depuration in the experiment (0, 1, 3, 6, 9, and 12). Three scallops from the first group were used to determine the initial (day 0) concentration of domoic acid (DA). The remaining groups were distributed among 15-L plastic containers filled with filtered seawater (1 µm) and continuous aeration. The containers were maintained at 16 ± 1 °C in a temperature-controlled walk-in environmental chamber. Each group of scallops was fed twice a day with *Isochrysis galbana* at a final density of 600,000 cells mL^−1^, which represents, approximately, a biovolume of 120 mm^3^. Water was replaced daily with clean seawater to minimize re-ingestion of feces. On days 1, 3, 6, 9, and 12, three scallops corresponding to the assigned group were sampled for dissection and toxin analysis.

### 5.2. Depuration of Domoic Acid in Natural Environment

Scallop samples were maintained in the usual culture conditions in scallop culture lanterns at the facilities of Invertec Ostimar Company located in a farm in Bahía Tongoy, Chile. Scallops of and approximated valve height of 7 cm (which finally resulted in an average of 7.15 ± 1.2) were sampled every other day from 2 to 14 August 2017 (days 0, 2, 4, 6, 8, 10, 12). Each sample consisted of 3–5 individuals which were processed as in the previous experiment. In this experiment, the population of the DA producer species *Pseudo-nitzschia australis* was substantially reduced, but still present at low concentrations (Table S1. Supplementary Materials). Consequently, some low level DA uptake likely continued.

During the period in which the experiment developed, data of abundance of *Pseudo-nitzschia*, and the main phytoplankton species present, were obtained weekly by the Molluscan Shellfish Safety Programme (PSMB Programa de Sanidad de los Moluscos Bivalvos) of SERNAPESCA (www.sernapesca.cl) using a dividable hose sampler (0–10 m) and quantified by the method of Utermöhl [53]. Roughly, the biovolume of phytoplankton available for each scallop (assuming a clearance rate of 10 L h^−1^ [54] and computing the biovolumes following Olenina et al. [55]) ranged from 1000 to 7500 mm^3^ day^−1^ with an average of 3200. Additionally, water temperature was measured with a YSI 550A oxygen meter at 10-m depth. During the experiment the temperature ranged from 13 to 14 °C.

### 5.3. Scallop Dissection and Domoic Acid Analysis

To determine domoic acid (DA) concentration and toxin burden, each individual was carefully dissected into the edible tissues corresponding to the gonad plus foot (Go), adductor muscle plus kidneys (Mu), and the non-edible tissues, corresponding to the digestive gland (DG), mantle (Ma), and gills (Gi). Domoic acid was extracted from scallop tissues following the procedure described by Quilliam, et al. [56], while matrix effects and extraction efficiency were evaluated following the method described by Regueiro et al. [57] (for result see Table S2, Supplementary Materials). Extraction was performed from approximately 4 g of tissue homogenate with 16 mL of MeOH/water (1:1, *v*/*v*) using an Ultra-Turrax T25 dispersing system (IKA^®^Werke GmbH & Co. KG, Staufen, Germany) at 11,000 rpm for 3 min. The extract was clarified by centrifugation at 5000× *g* for 20 min (Centurion K2015R, Centurion Scientific Ltd., Stoughton, West Sussex, UK). A one-mL aliquot was filtered through a 0.2 µm Clarinert nylon syringe filter (13 mm diameter) (Agela Technologies, Torrance, CA, USA) and stored in an autosampler vial at −20 °C until analysis.

The analysis was performed by LC-HRMS following the method described by Regueiro et al. [57] with modifications. The instrumental analysis was developed using a Dionex Ultimate 3000 UHPLC system (Thermo Fisher Scientific, Sunnyvale, CA, USA). A reversed-phase HPLC column Kinetex C18 (50 mm × 2.1 mm; 2.6 μm) with an Ultra Guard column C18 both from Phenomenex (Torrance, CA, USA) was used. The flow rate was set to 0.20 mL min^−1^, and the injection volume was 10 μL. Mobile phases A and B were water and MeOH/water (50/50, *v*/*v*), respectively, both containing 0.2% formic acid (pH = 2.4). The following gradient was used to achieve the chromatographic separation: 100% phase A for 2 min, decreased to 45% A over 2 min, hold at 45% A for 3.4 min, and then returned to initial conditions over 4.6 min. The total analysis run time was 12 min. The detection of domoic acid was carried out by a high-resolution mass spectrometer Q Exactive Focus equipped with an electrospray interphase HESI II (Thermo Fisher Scientific, Sunnyvale, CA, USA). The interface was operated in positive ionization mode with a spray voltage of 3.5 kV. The temperature of the ion transfer tube and the HESI II vaporizer were set at 250 °C. Nitrogen (>99.98%) was employed as sheath gas and auxiliary gas at pressures of 20 and 10 arbitrary units, respectively. The data were acquired in selected ion monitoring (SIM) and data-dependent (ddMS^2^) acquisition modes (for quantification and confirmation, respectively). In SIM mode, the mass was set to 312.1445 *m*/*z*, using a mass resolution of 70,000. DA was quantified by external calibration, using a DA certified reference solution (CRM-DA-g) (NRC, CNRC, Canada). The limit of quantification of the method was 2 ng mL^−1^ and the recoveries ranged from 97 to 117%. Some DA isomers, with retention times different from DA were also detected by this method but because of their limited toxicological interest and low transformation to or from DA (Tables S3 and S4, Supplementary Materials) they have not been included in this study.

### 5.4. Modelling

Two models were fitted to the obtained data. The simplest one, Model I (1) was a first-order exponential decay of domoic acid burden in each organ.

(1) $$\frac{d\left(DA_{0}\right)}{dt}=-DR_{0}\times DA_{0}$$

where *DA_o_* is the domoic acid burden in each organ (o), DR_o_ the depuration rate corresponding to the organ *o*, and *t* is the depuration time.

The second model used, Model II (2) also assumed a first-order exponential depuration in each organ, but it allows for toxin transfers from the digestive gland to all other studied organs.

(2) $$\frac{d\left(DA_{Dg}\right)}{dt}=-{\mathrm{DR}}_{\mathrm{Dg}}\times DA_{Dg}-{\Sigma \mathrm{TR}}_{\mathrm{Dg}-0}\times DA_{Dg}+K\cdot {\mathrm{Cells}}_{t}$$

$$\frac{d\left(DA_{Go}\right)}{dt}=-{\mathrm{DR}}_{\mathrm{Go}}\times DA_{Go}-{\mathrm{TR}}_{\mathrm{Dg}-\mathrm{Go}}\times DA_{Dg}$$

$$\frac{d\left(DA_{Ma}\right)}{dt}=-{\mathrm{DR}}_{\mathrm{Ma}}\times DA_{Ma}-{\mathrm{TR}}_{\mathrm{Dg}-\mathrm{Ma}}\times DA_{Dg}$$

$$\frac{d\left(DA_{Gi}\right)}{dt}=-{\mathrm{DR}}_{\mathrm{Gi}}\times DA_{Gi}-{\mathrm{TR}}_{\mathrm{Dg}-\mathrm{Gi}}\times DA_{Dg}$$

$$\frac{d\left(DA_{Mu}\right)}{dt}=-{\mathrm{DR}}_{\mathrm{Mu}}\times DA_{Mu}-{\mathrm{TR}}_{\mathrm{Dg}-\mathrm{Mu}}\times DA_{Dg}$$

where subindices denote the organ (Dg = digestive gland, Go = gonad, Ma = mantle, Gi = gill, and Mu = adductor muscle, and o = each organ other than digestive gland), TR the transfer rates, DR the depuration rates, Cells_t_ the toxic cell concentration in water at time t, and K, a proportionality coefficient to transform cell concentration into absorbed toxin. The models were implemented and fitted to the data using the R packages deSolve [58] and FME [59].

## Figures and Tables

**Table 1:** Parameters of the fitted models which included transfer from the digestive gland to other organs.

Organ	Natural Conditions		Laboratory Conditions	
	Depuration Rate	Transfer Rate	Depuration Rate	Transfer Rate
DG	0.03806		0.095	
Gonad	0.229144	0.06657	3.272	0.336
Mantle	0.524622	0.10365	0.504	0.005
Gill	1.424803	0.24322	4.202	0.048
Muscle	0.33993	0.1412	4.201	0.286

## Supplementary Materials

The following are available online at https://www.mdpi.com/2072-6651/12/11/698/s1, Figure S1: Logarithmically transformed domoic acid burden in the organs of *Argopecten purpuratus* during depuration experiment in laboratory conditions. Dots are the observations, triangles are the means, and the straight line is the regression line fitted to the transformed data. The equation of the line and its corresponding R^2^ are given at the top of each panel. The slopes of the regression lines are the depuration rate, assuming a first-order exponential depuration for each organ. Figure S2: Logarithmically transformed domoic acid burden in the organs of *Argopecten purpuratus* during the first three days of the depuration experiment in laboratory conditions. Dots are the observations, triangles are the means, and the straight line is the regression line fitted to the transformed data. The equation of the line and its corresponding R^2^ are given at the top of each panel. The slopes of the regression lines are the depuration rate, assuming a first-order exponential depuration for each organ. Figure S3: Logarithmically transformed domoic acid burden in the organs of *Argopecten purpuratus* during the depuration experiment in natural conditions. Dots are the observations, triangles are the means, and the straight line is the regression line fitted to the transformed data. The equation of the line and its corresponding R^2^ are given at the top of each panel. The slopes of the regression lines are the depuration rate, assuming a first-order exponential depuration for each organ. Figure S4: Logarithmically transformed domoic acid concentration in the organs of *Argopecten purpuratus* during the depuration experiment in natural conditions. Dots are the observations, triangles are the means, and the straight line is the regression line fitted to the transformed data. The equation of the line and its corresponding R^2^ are given at the top of each panel. The slopes of the regression lines are the depuration rate, assuming a first-order exponential depuration for each organ. Figure S5: Multi-compartment model fit to the toxin burden data obtained in the domoic acid depuration experiment in natural conditions. Small dots are the obtained data, large dots are their means, and lines are the output of the model. The model assumes that depuration takes place from the digestive gland and the kidney, after transfer of the toxin from the digestive gland to all other organs, and from those organs to the kidney. The toxin amount in the kidney and the adductor muscle were measured together (“Adductor muscle”). Figure S6. Selected chromatogram of extract of digestive gland containing domoic acid (DA) and its isomers ISO D, ISO A, and epi DA. Table S1: *Pseudo-nitzschia australis* concentration in the area during depuration experiment in natural conditions. The data were obtained from Invertec Ostimar and correspond to the same bay where the experiment was carried out but not to the precise location where the experiment was carried out. Table S2. Recovery of domoic acid and matrix effect in different tissues of *Argopecten purpuratus*. Table S3. Proportions of domoic acid (DA) and its isomers on selected days during depuration in laboratory experiment. Table S4. Proportions of domoic acid (DA) and its isomers on selected days during depuration in natural environment experiment.
