# Supplementary material for: Rapid Domoic Acid Depuration in the Scallop Argopecten purpuratus and Its Transfer from the Digestive Gland to Other Organs
Source: Toxins (Basel). 2020 Nov 3;12(11):698. doi: 10.3390/toxins12110698 (PMC7692689; doi:10.3390/toxins12110698)
Supplement: Supplementary file 1 [file toxins-12-00698-s001.pdf]

Supplementary Materials: Rapid Domoic Acid Depuration in the Scallop *Argopecten purpuratus* and Its Transfer from the Digestive Gland to Other Organs

Gonzalo Álvarez, José Rengel, Michael Araya, Francisco Álvarez, Roberto Pino, Eduardo Uribe, Patricio A. Díaz, Araceli E. Rossignoli, Américo López-Rivera and Juan Blanco

Table S1. *Pseudo-nitzschia australis* concentration in the area during depuration experiment in natural conditions. The data were obtained from Invertec Ostimar and correspond to the same bay where the experiment was carried out but not to the precise location where the experiment was carried out.

Day of Experiment	Cells L^{-1}
0	52767
2	49698
5	26235
12	17500

Table S2. Recovery of domoic acid and matrix effect in different tissues of *Argopecten purpuratus*.

	% Recovery	% RSD. *n* = 3
	30 ng mL^{-1}	
Gonad	117	4
Mantle	105	7
Gill	100	2
Digestive Gland	97	4
Muscle	103	1

Table S3. Proportions of domoic acid (DA) and its isomers on selected days during depuration in laboratory experiment.

	Day	% DA	% ISO D	% ISO A	% Epi DA
Digestive gland	1	89.2	5.4	4.5	0.9
	6	91.6	4.2	4	0.2
	9	94.8	4.3	0.9	0
Gonad	1	85.5	5.2	8.7	0.6
	6	89	2.9	4.7	3.4
	9	97.2	2.8	0	0
Muscle	1	89.5	5.1	4.9	0.5
	6	92.6	4.3	3.1	0
	9	92.4	1.6	6	0
Mantle	1	89.8	5.3	4.2	0.7
	6	78.3	5.1	8.1	8.5
	9	87.1	6.6	6.3	0
Gills	1	89.7	5.2	4.6	0.5
	6	90.6	4.7	4.4	0.3
	9	89.2	5.5	4.9	0.4

Table S4. Proportions of domoic acid (DA) and its isomers on selected days during depuration in natural environment experiment.

	Day	DA	ISO D	ISO A	Epi DA
Digestive gland	0	94.6	5.4	0	0
	6	85.8	7.4	4.2	2.6

	10	83.8	5.5	6.6	4.1
Gonad	0	92.6	7.4	0	0
	6	78.2	7.1	9.4	5.3
	10	86.6	5.4	4	4
Muscle	0	97.1	2.9	0	0
	6	82.3	7.2	6	4.5
	9	82.5	4.6	6.2	6.7
Mantle	0	89.3	6.6	4.1	0
	6	81.6	7.9	6	4.5
	9	81.1	5.4	7.2	6.3
Gills	0	89.2	6.2	4.6	0
	6	82.2	7.7	5.8	4.3
	9	82.6	4.6	6.2	6.6

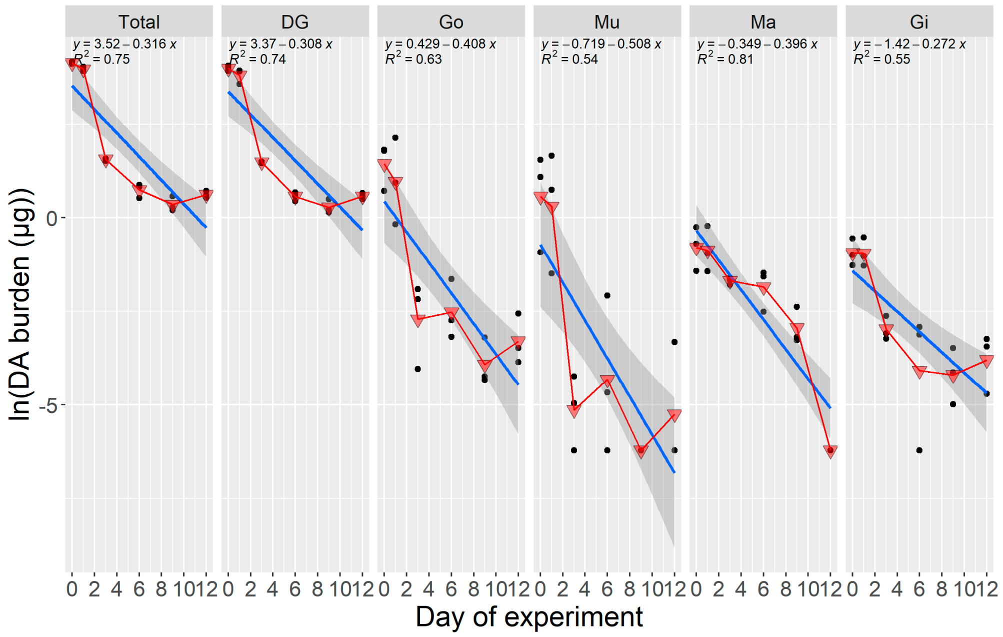

Figure S1. Logarithmically transformed domoic acid burden in the organs of *Argopecten purpuratus* during depuration experiment in laboratory conditions. Dots are the observations. triangles are the means. and the straight line is the regression line fitted to the transformed data. The equation of the line and its corresponding R^2 are given at the top of each panel. The slopes of the regression lines are the depuration rate. assuming a first order exponential depuration for each organ. Total = all soft tissues, DG = digestive gland, Go =gonad + foot, Mu = adductor muscle + kidneys, Ma = mantle, Gi = gills.

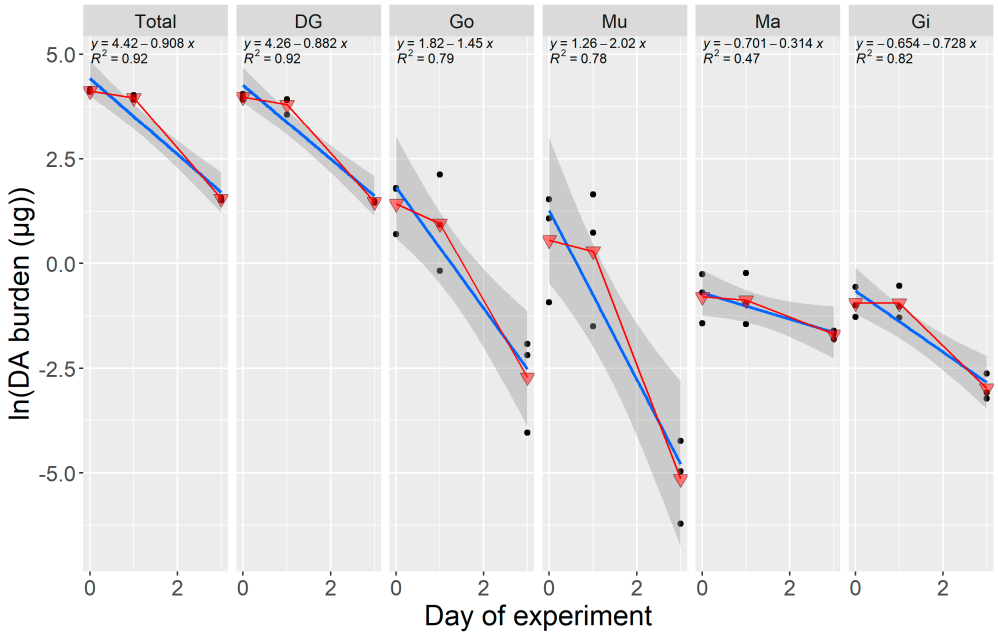

Figure S2. Logarithmically transformed domoic acid burden in the organs of *Argopecten purpuratus* during the first three days of the depuration experiment in laboratory conditions. Dots are the observations. triangles are the means. and the straight line is the regression line fitted to the transformed data. The equation of the line and its corresponding R^2 are given at the top of each panel. The slopes of the regression lines are the depuration rate. assuming a first order exponential depuration for each organ. Total = all soft tissues, DG = digestive gland, Go =gonad + foot, Mu = adductor muscle + kidneys, Ma = mantle, Gi = gills.

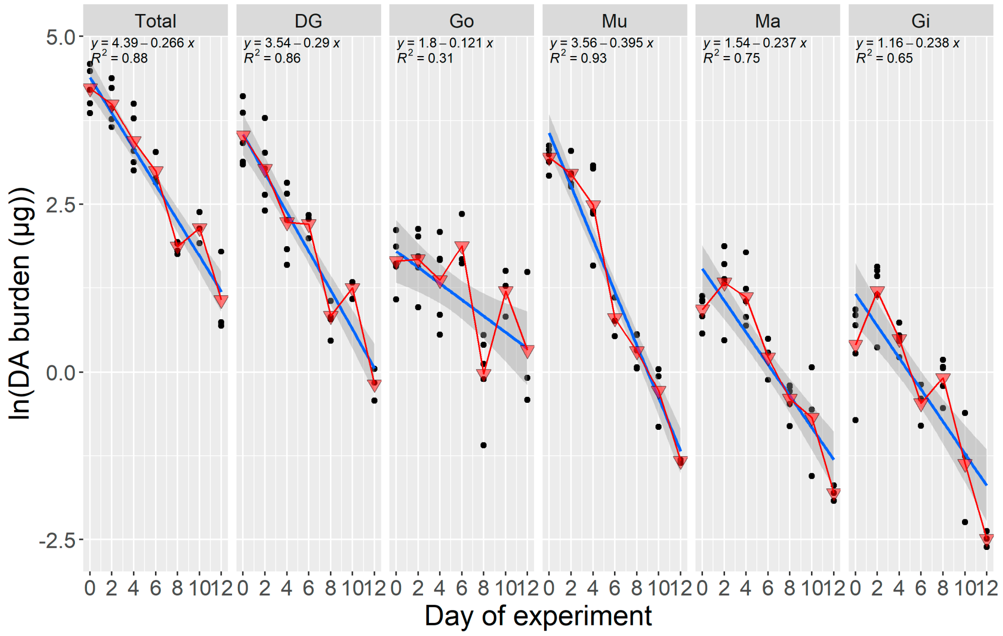

Figure S3. Logarithmically transformed domoic acid burden in the organs of *Argopecten purpuratus* during the depuration experiment in natural conditions. Dots are the observations. triangles are the means. and the straight line is the regression line fitted to the transformed data. The equation of the line and its corresponding R^2 are given at the top of each panel. The slopes of the regression lines are the depuration rate. assuming a first order exponential depuration for each organ. Total = all soft tissues, DG = digestive gland, Go =gonad + foot, Mu = adductor muscle + kidneys, Ma = mantle, Gi = gills.

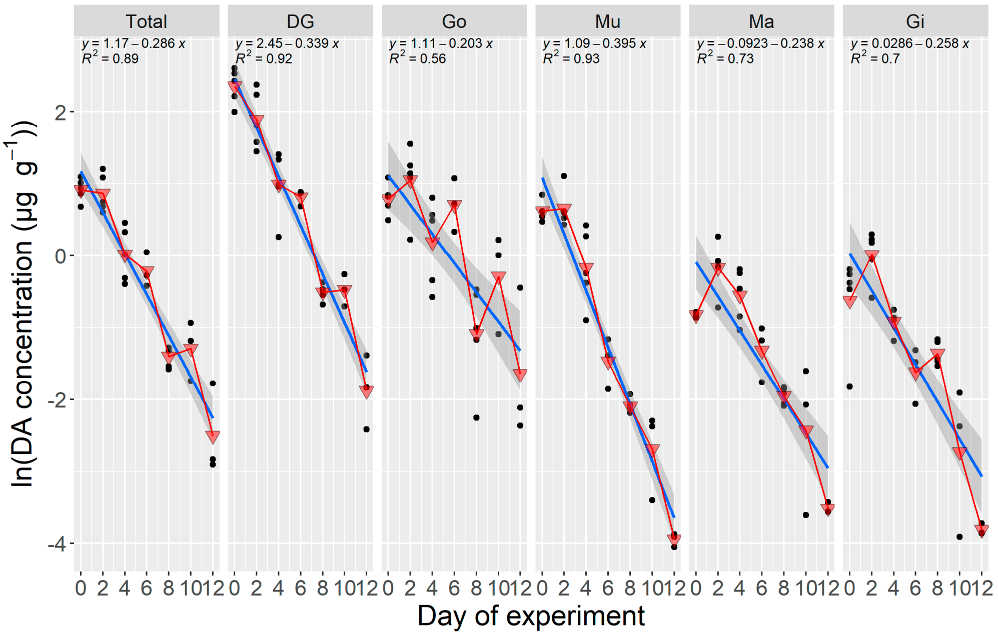

Figure S4. Logarithmically transformed domoic acid concentration in the organs of *Argopecten purpuratus* during the depuration experiment in natural conditions. Dots are the observations, triangles are the means, and the straight line is the regression line fitted to the transformed data. The equation of the line and its corresponding R^2 are given at the top of each panel. The slopes of the regression lines are the depuration rate, assuming a first order exponential depuration for each organ. Total = all soft tissues, DG = digestive gland, Go =gonad + foot, Mu = adductor muscle + kidneys, Ma = mantle, Gi = gills.

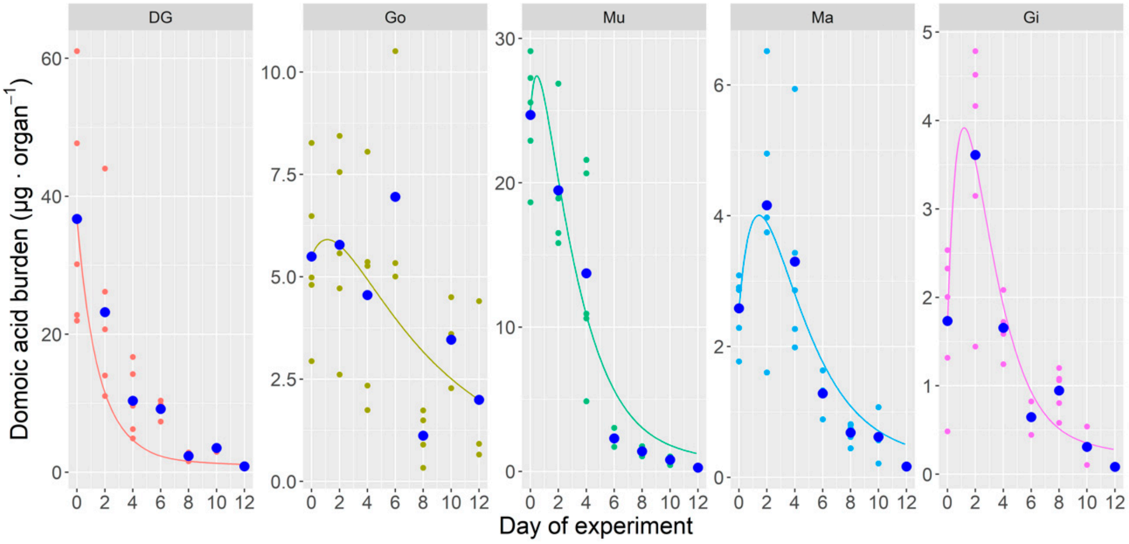

Figure S5. Multi-compartment model fit to the toxin burden data obtained in the domoic acid depuration experiment in natural conditions. Small dots are the obtained data, large dots are their means, and lines are the output of the model. The model assumes that depuration takes place from the digestive gland and the kidney, after transfer of the toxin from the digestive gland to all other organs, and from those organs to the kidney. The toxin amount in the kidney and the adductor muscle were measured together (“Adductor muscle”). DG = digestive gland, Go =gonad + foot, Mu = adductor muscle + kidneys, Ma = mantle, Gi = gills.

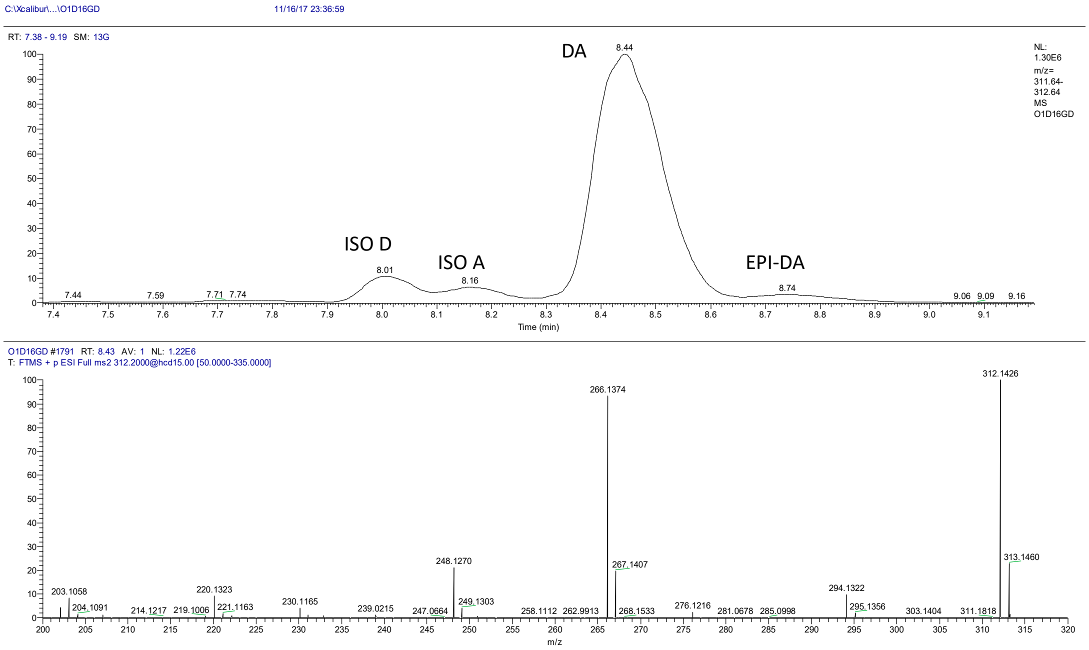

Figure S6. Selected chromatogram of extract of digestive gland containing domoic acid (DA) and its isomers ISO D, ISO A and epi DA.
